# AI Chatbot Use and Disclosure for Mental Health Among US Adolescents and Young Adults

**DOI:** 10.1001/jamapediatrics.2026.2015

**Published:** 2026-06-01

**Authors:** Ryan K. McBain, Jonathan H. Cantor, Joshua Breslau, Melissa Diliberti, Li Ang Zhang, Fang Zhang, Alyssa Burnett, Aaron Kofner, Benjamin Rader, Pat Pataranutaporn, Bradley D. Stein, Ateev Mehrotra, Hao Yu

**Affiliations:** 1 RAND, Arlington, Virginia; 2 Mass General Brigham, Boston, Massachusetts; 3 Harvard Medical School, Boston, Massachusetts; 4 RAND, Santa Monica, California; 5 RAND, Pittsburgh, Pennsylvania; 6 Harvard Pilgrim Health Care Institute, Boston, Massachusetts; 7 Boston Children’s Hospital, Boston, Massachusetts; 8 MIT Media Lab, MIT, Cambridge, Massachusetts; 9 Brown University School of Public Health, Providence, Rhode Island

## Abstract

**Importance:**

The rapid expansion of artificial intelligence (AI) chatbots has coincided with a persistent youth mental health crisis in the US, raising a question about the extent to which young people are turning to this technology for mental health advice.

**Objective:**

To assess the prevalence, frequency, perceived helpfulness, and disclosure of AI chatbot use for mental health advice among US adolescents and young adults in 2025.

**Design, Setting, and Participants:**

This cross-sectional, nationally representative survey was conducted with adolescents and young adults aged 12 to 21 years in November 2025.

**Exposures:**

Exposures included self-reported age, sex, race and ethnicity, census region, metropolitan status, and prior discussion with a clinician about mental health in the past 6 months.

**Main Outcomes and Measures:**

Self-reported use of AI chatbots for mental health advice, including any prior use, frequency of use, perceived helpfulness of responses, and disclosure of use to others. Respondents were also asked whether they had spoken with a physician about their mental health in the prior 6 months. Using multivariable logistic regression analysis, variation in responses was assessed according to respondents’ demographic and geographic characteristics.

**Results:**

Among a US population-weighted 42 825 655 youth (unweighted, 1009 youth; median [IQR] age, 17 [15-18] years; population-weighted 21 410 663 male [50.0%]), 19.2% of adolescents and young adults (population-weighted n = 8 207 180) in 2025 reported having used AI chatbots for mental health advice. Among those who sought advice from AI chatbots, 42.8% did so at least monthly, and 91.7% rated the advice as somewhat or very helpful. Most adolescents reported they had not disclosed AI chatbot use for mental health advice to anyone (63.3%). Use of an AI chatbot for mental health advice was more common among females compared with males (adjusted odds ratio [aOR], 2.10; 95% CI, 1.36-3.23), respondents aged 18 to 21 years compared with respondents aged 12 to 14 years (aOR, 3.65; 95% CI, 1.98-6.74), and those who had spoken with a physician about their mental health in the prior 6 months compared with those who had not (aOR, 1.89; 95% CI, 1.18-3.03).

**Conclusions and Relevance:**

In this nationally representative survey study of US adolescents and young adults, a fifth reported using AI chatbots for mental health advice. AI chatbots are already embedded in many youths’ mental health information ecosystem, underscoring the need for parents and clinicians to proactively discuss chatbot use to promote safety, appropriate expectations, and linkages to evidence-based care.

## Key Points

**Questions:**

As of 2025, what percentage of US adolescents and young adults aged 12 to 21 years used artificial intelligence (AI) chatbots for mental health advice, and among those who use AI chatbots for this purpose, to what extent did they tell others?

**Findings:**

This national survey including more than 42 million US youth (population-weighted) found that almost a fifth of adolescents and young adults reported using AI chatbots for mental health advice, representing an increase by almost half from 1 year prior. Most users told no one that they used AI chatbots for this purpose.

**Meaning:**

AI chatbots are widely used by adolescents and young adults for emotional and psychological support, underscoring the urgent need for parents, clinicians, and policymakers to understand their evolving role in youth mental health care.

## Teaser

This survey study evaluates the percentage of US adolescents and young adults who used artificial intelligence (AI) chatbots for mental health advice as of 2025 and to what extent they told others.

## Introduction

Since the commercial launch of artificial intelligence (AI) chatbots such as ChatGPT (OpenAI), awareness and adoption have been swiftest among adolescents and young adults.^1^ A 2025 Pew Research survey found that 64% of teens aged 13 to 17 years reported having ever used an AI chatbot, and 28% use them daily.^2^ During the same period, the US has been confronting a mental health crisis among adolescents and young adults.^3^ Suicide is the second leading cause of death among those aged 5 to 24 years.^4,5^ A Centers for Disease Control and Prevention (CDC) survey in 2023 found that 1 in 5 high schoolers (20.4%) considered attempting suicide, and almost 1 in 10 (9.5%) reported ever attempting suicide.^6^ Rates of depression (18%) and anxiety disorders (20%) among adolescents are alarmingly high.^7^ Nevertheless, 4 in 10 teenagers with a major depressive episode in the past year report not receiving mental health services,^8^ and anxiety disorders have an even larger treatment gap.^9^

The rapid adoption of AI chatbots raises the question of how often adolescents and young adults turn to them for mental health advice.^10,11,12^ Studies conducted before the introduction of large language model (LLM)–based AI chatbots have found that adolescents frequently search for mental health information online, in part because the internet is easily accessible, anonymous, and low-cost.^13^ AI chatbots could be even more attractive as sources of advice because they offer personalized and interactive guidance.^14^ These same features raise concerns, as exchanges occur without oversight by adults or mental health professionals.^14,15,16^

In a 2024 national survey, we found that 1 in 8 adolescents and young adults reported using generative AI for mental health advice.^17^ In the current study, we examined prevalence, frequency of use, and perceived helpfulness in 2025, while expanding the question set to include disclosure of use to family and friends and whether AI chatbot use coincided with seeking mental health services from a physician. We also examined the extent to which these measures varied according to respondents’ demographic and geographic characteristics.

## Methods

### Study Design

This cross-sectional survey study received approval from Harvard Pilgrim Health Care Institute’s institutional review board. The study adheres to Strengthening the Reporting of Observational Studies in Epidemiology (STROBE) guidelines.

### Procedures

Participants were drawn from members of RAND’s American Life Panel (ALP), a nationally representative survey panel.^18^ ALP panelists regularly complete internet-based surveys on an array of topics, including health. Surveys are administered online, in English, and participants receive financial compensation. Adults 18 and older are initially enrolled based on random samples of household unit listings from the US Postal Service’s Delivery Sequence File. Adult panel members provided written informed consent to the participation of eligible minors within their household for surveys administered to those younger than 18 years; minors then provide assent. For this study, 1727 youth aged 12 to 21 years were invited to participate. We applied survey weights to account for US population demographics in terms of age, race and ethnicity (Black, Hispanic, non-Hispanic White, and other, which represents all categories apart from those classified), sex, and geographic region. Race and ethnicity information was used to inspect demographic differences on each outcome of interest. The survey was administered in November 2025.

### Measures

We used 6 survey questions on AI chatbot use for mental health advice. Four of the questions corresponded to those in a 2024 survey by the research team.^17^ Respondents were first informed that chatbots that answer questions—such as ChatGPT, Google Gemini (Google), My AI (Snap Inc), Character.AI (Character Technologies), and Meta AI (Meta)—are examples of AI chatbots. Respondents were then asked whether they have ever used an AI chatbot. Among those who responded yes, respondents reported whether they had ever asked an AI chatbot for advice about their mental health—specifically, when feeling sad, angry, nervous, or stressed (yes or no). Survey methodology is available in eMethods 1 in Supplement 1.

Respondents who reported ever using an AI chatbot for mental health advice then indicated their frequency of such use (hardly ever, at least once a month, at least once a week, daily or almost daily), perceived helpfulness (very helpful, somewhat helpful, or not helpful), and whether they had disclosed this use to anyone (eg, parent or guardian, brother or sister, friend). Lastly, we asked all survey respondents whether they had spoken about their mental health in the past 6 months to a clinician (yes or no). The full survey instrument is viewable in eMethods 2 in Supplement 1.

Individual demographic variables collected in both survey panels included sex, age, and race and ethnicity. We linked geographic identifiers for each respondent to their US census region and defined metropolitan or nonmetropolitan status based on ZIP code–level information and rural-urban commuting area codes specified by the US Department of Agriculture Economic Research Service.^19^

### Statistical Analysis

We first summarized responses to each question using survey-weighted percentages, then conducted cross-tabulations with demographic and geographic characteristics. We subsequently conducted multivariable logistic regression analyses, examining the association between survey responses and joint demographic and geographic characteristics previously described, as well as whether the individual had spoken to a physician in the prior 6 months about their mental health. The outcomes specified in regression analyses were as follows: (1) any AI chatbot use for mental health advice (yes or no), (2) monthly or more often frequency of AI chatbot use for mental health advice (yes or no), (3) AI chatbot used for mental health advice perceived as somewhat or very helpful (yes or no), and (4) disclosure of AI chatbot use for mental health advice (yes or no). These analyses incorporated survey weights, reported adjusted odds (each odds ratio for each explanatory variable was adjusted for all other explanatory variables in the model), and were performed using Stata, version 19.5 (StataCorp).

## Results

### Descriptive Characteristics

Of 1727 individuals contacted, 1009 responded to the survey. Unweighted and weighted sample characteristics are provided in Table 1. The US population-weighted sample included 42 825 655 youth (median [IQR] age, 17 [15-18] years; population-weighted 20 916 260 female [48.8%]; 21 410 663 male [50.0%]). In terms of race and ethnicity, the population-weighted sample included 5 563 350 Black (13.0%), 11 010 863 Hispanic (25.7%), 21 325 219 White (49.8%), and 4 316 881 other (10.1%) respondents.

**Table 1.** Descriptive Characteristics of Survey Sample

Sample characteristic	Sample size (%)	
	Unweighted	Weighted
Sex		
Male	427 (42.3)	21 410 663 (50.0)
Female	570 (56.5)	20 916 260 (48.8)
Missing^a^	12 (1.2)	498 732 (1.2)
Age, y		
12-14	232 (23.0)	8 560 172 (20.0)
15-17	455 (45.1)	16 950 151 (39.6)
18-21	322 (31.9)	17 315 332 (40.4)
Race and ethnicity		
Black	94 (9.3)	5 563 350 (13.0)
Hispanic	192 (19.0)	11 010 863 (25.7)
White non-Hispanic	647 (64.1)	21 325 219 (49.8)
Other^b^	68 (6.7)	4 316 881 (10.1)
Missing^a^	8 (0.8)	609 342 (1.4)
Census region		
Northeast	180 (17.8)	7 002 578 (16.4)
Midwest	230 (22.8)	9 006 184 (21.0)
South	385 (38.2)	16 648 986 (38.9)
West	214 (21.2)	10 167 908 (23.7)
Metropolitan status		
Not metropolitan	133 (13.2)	5 038 619 (11.8)
Metropolitan	876 (86.8)	37 787 036 (88.2)
Total	1009 (100.0)	42 825 655 (100.0)

^a^ Missing indicates that the survey respondent did not provide a response to the relevant question.

^b^ Other race and ethnicity represents all categories apart from those classified.

### Use of AI Chatbots

**Figure 1.**
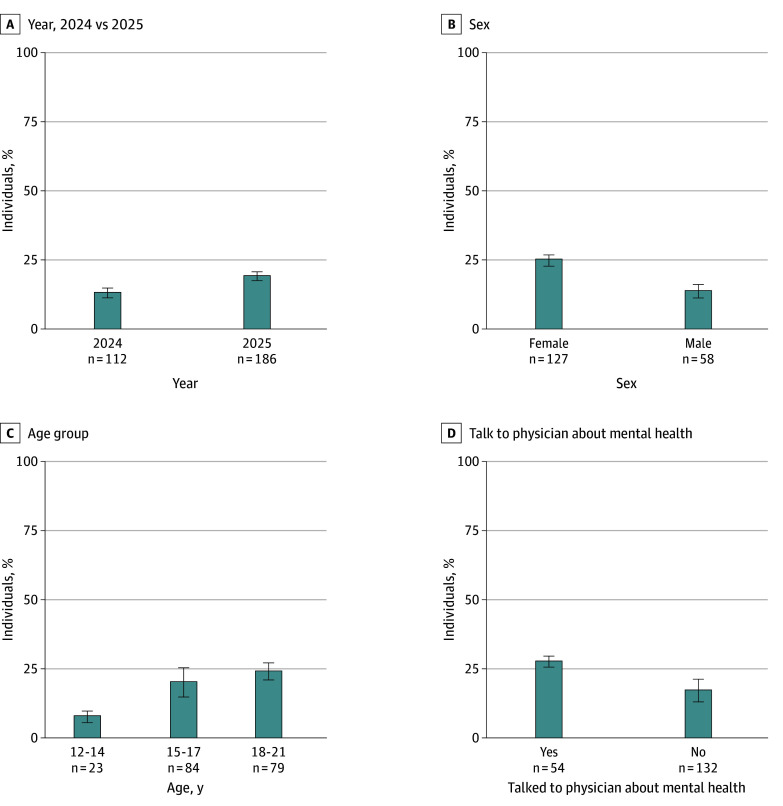
Reported Use of Artificial Intelligence (AI) Chatbots for Mental Health Advice Among Young People in the US Each bar represents the survey-weighted percentage of individuals within the subgroup who report that they have used an AI chatbot for mental health advice. Numbers represent the unweighted number of survey respondents, within the category, who report that they used an AI chatbot for mental health advice.

Applying survey weights to all responses, we found that almost 1 in 5 respondents (19.2%; population-weighted n = 8 207 180) stated they had ever used an AI chatbot for mental health advice. Usage was highest among those aged 18 to 21 years (24.1%), females (25.0%), and those who had spoken with their physician in the past 6 months about their mental health (27.7%) (Figure 1). Of those who had used AI chatbots for mental health advice, 42.8% did so at least monthly, 26.3% reported use at least once a month, 10.8% at least once a week, and 5.8% daily or almost daily. In terms of helpfulness, 91.7% rated the advice as somewhat or very helpful (25.0% found the advice to be very helpful and 66.7% found it somewhat helpful), and 8.3% found it not helpful. Additionally, 21.1% of respondents reported discussing their mental health with a physician in the prior 6 months.

Most respondents who used an AI chatbot for mental health advice had not disclosed this to anyone (63.3%). Those who disclosed use were most likely to disclose this to a friend (28.0%), or a trusted adult such as a parent, teacher, or physician (16.4%).

### Respondent Characteristics Associated With AI Chatbot Mental Health Use

Use of an AI chatbot for mental health advice was more common among females compared with males (adjusted odds ratio [aOR], 2.10; 95% CI, 1.36-3.23), among older respondents compared with those aged 12 to 14 years (aged 15-17 years: aOR, 2.84; 95% CI, 1.53-5.28; aged 18-21 years: aOR, 3.65; 95% CI, 1.98-6.74), among respondents who listed their race and ethnicity as other (aOR, 2.50; 95% CI, 1.22-5.13) compared with those who listed their race as White, and among respondents who had spoken with a physician about their mental health in the past 6 months (aOR, 1.89; 95% CI, 1.18-3.03) compared with those who had not. We did not find significant differences with respect to metropolitan status, or census region (Table 2).

**Table 2.** Correlates of Artificial Intelligence (AI) Chatbot Use for Mental Health Advice Among Adolescents and Young Adults in the US

Independent measure	Outcome 1; outcomes 2-4, No.	Ever asked chatbot for mental health advice		Asked chatbot for mental health advice monthly or more often		AI chatbot mental health advice perceived as helpful		Disclosure of using AI chatbot for mental health advice	
		aOR^a^ (95% CI)	*P* value	aOR^a^ (95% CI)	*P* value	aOR^a^ (95% CI)	*P* value	aOR^a^ (95% CI)	*P* value
Sex									
Female	n = 569; n = 127	2.10 (1.36-3.23)	.001	1.16 (0.53-2.58)	.71	1.24 (0.33-4.75)	.75	0.60 (0.26-1.37)	.23
Male	n = 423; n = 57	1 [Reference]	Ref	1 [Reference]	Ref	1 [Reference]	Ref	1 [Reference]	Ref
Age group, y									
12-14	n = 229; n = 23	1 [Reference]	Ref	1 [Reference]	Ref	1 [Reference]	Ref	1 [Reference]	Ref
15-17	n = 448; n = 83	2.84 (1.53-5.28)	.001	2.45 (0.65-9.27)	.19	0.42 (0.07-2.36)	.32	0.76 (0.22-2.63)	.66
18-21	n = 315; n = 78	3.65 (1.98-6.74)	<.001	2.32 (0.59-9.08)	.23	0.56 (0.09-3.30)	.52	0.81 (0.23-2.84)	.74
Race and ethnicity									
Black	n = 94; n = 16	0.79 (0.40-1.55)	.49	5.45 (1.44-20.66)	.01	NA^b^	NA^b^	0.57 (0.18-1.81)	.34
Hispanic	n = 189; n = 38	1.27 (0.75-2.15)	.38	2.20 (0.93-5.20)	.07	4.70 (0.42-52.62)	.21	0.46 (0.18-1.18)	.10
White non-Hispanic	n = 641; n = 113	1 [Reference]	Ref	1 [Reference]	Ref	1 [Reference]	Ref	1 [Reference]	Ref
Other^c^	n = 68; n = 17	2.50 (1.22-5.13)	.01	1.83 (0.53-6.29)	.34	0.89 (0.12-6.80)	.91	0.33 (0.09-1.16)	.08
Metropolitan status									
Not metropolitan	n = 132; n = 22	1 [Reference]	Ref	1 [Reference]	Ref	1 [Reference]	Ref	1 [Reference]	Ref
Metropolitan	n = 860; n = 162	1.09 (0.57-2.08)	.80	2.42 (0.71-8.28)	.16	0.18 (0.02-1.59)	.12	0.52 (0.13-2.02)	.34
Census region									
South	n = 380; n = 71	1 [Reference]	Ref	1 [Reference]	Ref	1 [Reference]	Ref	1 [Reference]	Ref
Northeast	n = 177; n = 40	1.02 (0.59-1.78)	.93	0.94 (0.34-2.57)	.90	0.43 (0.10-1.93)	.27	1.33 (0.45-4.00)	.61
Midwest	n = 228; n = 37	0.70 (0.39-1.26)	.24	0.38 (0.13-1.13)	.08	0.60 (0.09-4.24)	.61	0.50 (0.17-1.48)	.21
West	n = 207; n = 36	0.87 (0.49-1.55)	.63	0.73 (0.28-1.94)	.53	0.75 (0.14-4.09)	.74	1.33 (0.49-3.63)	.58
Talked to physician^d^									
No	n = 768; n = 130	1 [Reference]	Ref	1 [Reference]	Ref	1 [Reference]	Ref	1 [Reference]	Ref
Yes	n = 224; n = 54	1.89 (1.18-3.03)	.008	0.71 (0.29-1.72)	.45	0.40 (0.13-1.29)	.12	1.01 (0.43-2.35)	.99

Abbreviations: aOR, adjusted odds ratio, NA, not available; ref, reference.

^a^ aOR represents adjusted odds ratios from multivariable logistic regression including all independent measures.

^b^ Missing values for the third outcome are because the category perfectly predicted the outcome (complete separation), yielding nonfinite coefficient estimates.

^c^ Other race and ethnicity represents all categories apart from those classified.

^d^ Represents having talked to a physician in the prior 6 months about one’s mental health.

### Correlates of AI Chatbot Use Behaviors

**Figure 2.**
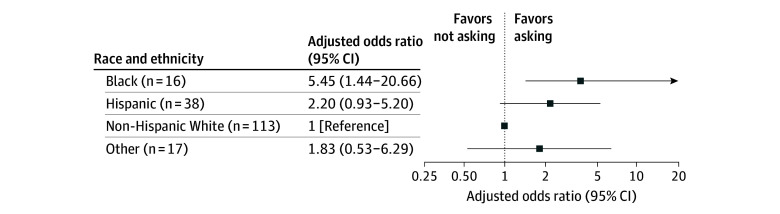
Adjusted Odds of Asking Artificial Intelligence (AI) Chatbots for Mental Health Advice Monthly or More Often Data from weighted US adolescent and young adult survey; multivariable model adjusts for sex, age, metro status, region, and physician discussion on mental health needs in prior six months. Numbers represent the number of respondents in each race and ethnicity category. Other race and ethnicity represents all categories apart from those classified.

Those reporting AI chatbot use for mental health advice at least monthly were more likely to be Black (aOR, 5.45; 95% CI, 1.44-20.66) than White (Figure 2). We did not find statistically significant differences in frequency of use with respect to sex, age, metropolitan status, census region, or having spoken with a physician about mental health in the past 6 months. With respect to perceived helpfulness and disclosure, we did not find any statistically significant differences among explanatory variables of interest.

## Discussion

In this nationally representative survey, we estimated that roughly 1 in 5 adolescents and young adults in the US—representing approximately 8 million individuals—have ever used an AI chatbot for mental health advice as of 2025. This figure compares with 1 in 8 adolescents and young adults (13.1%) from a similar survey^17^ using slightly different wording conducted by our research team in 2024. Adolescents and young adults who have spoken to a physician about their mental health in the past 6 months were more likely to have used an AI chatbot for mental health advice. Among those using AI chatbots for mental health advice, the majority (approximately 5 million) did not disclose their use of AI chatbots for mental health advice to anyone.

Our results suggest that use of AI chatbots for mental health advice among adolescents and young adults is not a marginal phenomenon: 19.2% of respondents reported using AI chatbots for this purpose, a percentage similar in magnitude to the 19.8% receiving counseling from a mental health professional.^7^ These measures are not equivalent; counseling reflects formal clinical care, whereas our survey captures a broader range of engagements for mental health advice. Even so, the prevalence of chatbot use raises important questions about the role that AI may be playing in young people’s emotional lives.

On the one hand, the level of engagement among a subset of users—1 in 12 respondents reported using AI chatbots for mental health advice monthly or more often—suggests that AI chatbots may be filling a gap in emotional or psychological support. A majority of users also found AI chatbot responses somewhat or very helpful. On the other hand, perceived helpfulness may reflect AI chatbots’ tendencies toward sycophancy and overflattery, rather than the quality of advice they provide.^20,21^ Clinical experts have expressed concern that those with more intensive mental health needs may rely on therapeutic guidance from AI chatbots when such support cannot substitute for trained mental health professionals.^14^ AI chatbots specifically designed to offer therapeutic guidance—in contrast with general purpose AI chatbots—are still nascent, as are benchmarks for determining their overall performance,^22,23,24^ and there is limited transparency about the datasets used to train these models.^25^

Although mental health advice from AI chatbots may circumvent stigma associated with disclosure to adults or peers,^11,16,26^ this privacy also poses risks if individuals do not disclose their use of these tools to health care professionals or trusted adults. We found that almost two-thirds of adolescents and young adults (63.3%) who use AI chatbots for mental advice do not tell anyone. This finding is consistent with previous qualitative research showing that adolescent use of AI is happening without parental knowledge or involvement.^27^ To our knowledge, there are no directly comparable studies quantifying the extent to which young people disclose to parents their use of traditional, human-delivered mental health care. However, a recent CDC survey found substantial parent-teen concordance regarding their adolescents’ receipt of medical care more broadly,^28^ suggesting that parental awareness may be higher in conventional care settings than in the context observed here. Without disclosure by young people on the role of AI in providing mental health advice, clinicians may also be unaware of potentially influential or inaccurate advice provided by these tools to their patients, missing opportunities to offer guidance, context, or monitoring. Parents, clinicians, and educators should consider asking adolescents and young adults about their engagement with AI chatbots and provide counsel on their strengths and limitations.

Use of AI chatbots for mental health advice was more common in older adolescents and young adults, paralleling greater prevalence of mental health needs and conditions among this group.^9^ Use may also be more common in older respondents due to greater access to smartphones and reduced parental supervision of online activity. We also found that females were more likely to use AI chatbots for mental health advice, possibly because of a higher prevalence of mental health conditions.^29^ Lastly, we observed that—conditional on using AI chatbots for mental health advice—Black youth were over 5 times more likely than White youth to seek mental health advice monthly or more often. This may reflect a sense, within this population, that professionals are not as responsive to their unique needs, or else reflect reduced access to professional services.^30^ However, the sample size for this particular analysis was small, and follow-on investigation is warranted.

### Limitations

We note several key study limitations. First, we did not inquire about specific AI chatbots, and we did not examine heterogeneity of respondents’ experiences and outcomes. Both limit the specificity of our findings. Second, AI chatbots built on generative AI are constantly evolving; we present a snapshot as of late 2025. Third, the overall sample size was 1009, and the completion rate was 58.4%. Although this implies our findings contain uncertainty and may be subject to nonresponse bias, this is a common concern in surveys measuring use of AI.^31^ Additional bias may derive from the use of a web-based survey among English-speaking respondents, as these respondents may be more (or less) likely to use LLMs compared with other populations. Fourth, our investigation intentionally used language that would be accessible to individuals aged 12 to 21 years, such as “advice or help when you feel sad, angry, or nervous.” We did not inquire about mental health diagnoses, instead opting for more concrete language about individuals’ emotional experiences. Lastly, the survey does not aim to assess the quality of care delivered by AI chatbots, and this represents a critical area for future research. Despite these limitations, this survey study provides a novel snapshot into adolescents’ and young adults’ engagement with AI chatbots, offering insights on how these technologies are being used as sources of mental health advice.

## Conclusions

The finding of this nationally representative survey that almost 1 in 5 adolescents in the US reported use of AI chatbots for mental health advice—with over 40% of users doing so monthly or more often—underscores the urgency of understanding and shaping the evolving role of AI chatbots in youth mental health care. As these technologies become increasingly integrated into the daily lives of young people, they should be understood as active contributors in the broader ecosystem of psychological interventions.

## Notes

Supplement 1. **eMethods 1.** Survey Methodology **eMethods 2.** List of Questions

Supplement 2.
Data Sharing Statement.
