# Supplementary material for: AI Chatbot Use and Disclosure for Mental Health Among US Adolescents and Young Adults
Source: JAMA Pediatr. 2026 Jun 1;180(8):884–90. doi: 10.1001/jamapediatrics.2026.2015 (PMC13227335; doi:10.1001/jamapediatrics.2026.2015)
Supplement: Supplement 1. — eMethods 1. Survey Methodology eMethods 2. List of Questions [file jamapediatr-e262015-s001.pdf]

Supplementary Online Content

McBain RK, Cantor J, Breslau J, et al. AI chatbot use and disclosure for mental health among US adolescents and young adults. *JAMA Pediatr*. Published online June 1, 2026. doi:10.1001/jamapediatrics.2026.2015

eMethods 1. Survey Methodology

eMethods 2. List of Questions

This supplementary material has been provided by the authors to give readers additional information about their work.

© 2026 McBain RK et al. *JAMA Pediatrics*.

eMethods 1. Survey Methodology

Procedures

Participants were drawn from members of RAND's American Life Panel (RAND-ALP), which is a nationally representative survey panel of persons in the United States. [19] Unlike clinical-based samples, convenience samples, or samples of medical records, this panel was constructed using probability-based sampling methods applied to the universe of housing units in the U.S. Therefore, data from this panel can support inference to the online population of English-speaking U.S. youth between the ages of 12 and 21. RAND-ALP panelists regularly complete online surveys on an array of topics including education, employment, and health. Surveys are administered online, in English, and participants receive financial compensation. Adults aged 18 and older are initially enrolled in the RAND-ALP based on random samples of household unit listings from the U.S. Postal Service's Delivery Sequence File. Adult panel members consent to the participation of eligible minors within their household for surveys focused on youth.

For this study, 1,727 young adults were invited to participate, following parental consent and adolescent assent for respondents aged 17 or younger. The survey was administered between November 4, 2025, and November 19, 2025. 58% of panelists invited to take the survey completed it, which is in line with other major population-based health surveys. For context, the U.S. Centers for Disease Control and Prevention administers two national surveys to random samples of U.S. households: The National Health Interview Survey (NHIS) and the National Health and Nutrition Examination Survey (NHANES). These two surveys, which collect health condition and health behavior data, are used to produce official prevalence rates for the country. In the 2024 NHIS, response rates for adults and children were 48% and 46%, respectively. In the 2021–23 NHANES, response rates for 12- to 15-year-olds and 16- to 20-year-olds were 37% and 32%, respectively. We applied survey weights to our analytic sample to align with U.S. population distribution benchmarks of age, race/ethnicity, sex, and geographic region from the 2023 Current Population Survey.

eMethods 2. List of Questions

AI chatbots use artificial intelligence (AI) to talk with you and answer your questions. Examples of AI chatbots include ChatGPT, Google's Gemini, Snap's My AI, Character.AI, and Meta AI.

(1) **Have you ever used an AI chatbot?**

a. Yes (go to question 2)
b. No (go to question 6)

(2) **Have you ever asked an AI chatbot for advice about your mental health? By "mental health," we mean your thoughts and feelings—like being sad, angry, nervous, or stressed**

a. Yes (go to question 3)
b. (go to question 6)

(3) **<u>How often</u> do you ask an AI chatbot for advice about your mental health?**

a. Hardly ever
b. At least once a month
c. At least once a week
d. Daily or almost daily

(4) **How helpful is the AI chatbot when giving you advice about your mental health?**

a. Very helpful
b. Somewhat helpful
c. Not helpful

(5) **Have you <u>told any of the following people</u> that you used an AI chatbot for advice about your mental health?** Check all that apply.

a. Parent or guardian
b. Brother or sister
c. Friend
d. Teacher
e. Doctor or therapist
f. Someone else
g. No one

© 2026 McBain RK et al. *JAMA Pediatrics*.

(6) **In the past six months, have you seen a doctor for your mental health? This can include a psychologist, psychiatrist, or therapist.**
   a. Yes
   b. No—but I think it would be helpful
   c. No—I do not think it would be helpful

© 2026 McBain RK et al. *JAMA Pediatrics*.
