# Supplementary material for: AI Chatbot Use and Disclosure for Mental Health Among US Adolescents and Young Adults
Source: JAMA Pediatr. 2026 Jun 1;180(8):884–90. doi: 10.1001/jamapediatrics.2026.2015 (PMC13227335; doi:10.1001/jamapediatrics.2026.2015)
Supplement: Supplement 2. — Data Sharing Statement. [file jamapediatr-e262015-s002.pdf]

Data Sharing Statement

McBain. AI Chatbot Use and Disclosure for Mental Health Among US Adolescents and Young Adults. *JAMA Pediatr*. Published June 01, 2026. doi:10.1001/jamapediatrics.2026.2015

Data
Data available: Yes
Data types: Data dictionary
How to access data: Data dictionary available upon request from the study's corresponding author. The full dataset may be available upon request if inquirers contact RAND's American Youth Panel purveyors.
When available: With publication

Supporting Documents
Document types: None

Additional Information
Who can access the data: Anyone requesting the data
Types of analyses: For any purpose
Mechanisms of data availability: After approval of a proposal
Any additional restrictions: N/A
